# The King and the Hospitals

**Published:** 1901-02-09

**Authors:** 


### The Institutional Workshop.

### THE KING AND THE HOSPITALS.

No one could have watched the career of the King during the long time that he was Prince of Wales, and noted how, instead of merely sitting still and waiting for his Kingdom, he busied himself with the affairs of his people, and interested himself with the doings of every grade of society, without admitting that, whatever else may be said in his praise or his disfavour, at least this must be granted, that he has shown himself a many-sided man. People have said that the Prince required to be constantly amused. Perhaps it would be more true to say that he required to be constantly interested, and verily his capacity for taking interest has sometimes seemed insatiable. Some people, doubtless, have been puzzled, and not a few have been quite unable to understand, such many-sidedness. That the same man should bet and race and go to church; that the central figure of a society which cares little for anything beyond the moment should be the patron of hospitals and charities, of scientific bodies and of evangelising schemes; and that he should not only be the patron but should be thoroughly interested in them all, should attend their meetings, and should show from his place in the chair that he knew all about them—these were things that some people could never understand, any more than they could comprehend the human feelings which underlay all the actions of the Prince. We are apt to put princes on high pedestals and to forget that they are men. No one could be more a prince than the Prince of Wales, but no one more than he could be mere man with purely human feelings in moments of sorrow and of trouble. For he has not been without sorrow; and those who recall the time of the illness of the Duke of Clarence, the days of nursing and anxiety, and remember how when all was over he trudged like any peasant through slush and snow behind the coffin of his dead son, will recognise how strongly developed within him are those human feelings which form a bond of union between this many-sided prince and the many-sided people over whom he now rules. We need hardly be surprised then that among the multitude of affairs demanding the attention of the Prince the hospitals should have claimed a share. But if one traces from year to year the work which the Prince did, each year it becomes more evident that his work in this direction was not done from mere sense of duty or of fitness, but because by degrees hospital questions came to absorb a larger and larger share of his interest and attention until they became almost a hobby. Thus it came to pass that in the public eye the name of the Prince of Wales became inseparably linked with hospital affairs and with questions of the improvement of the hospital system in London. It is possible that the fact that in early life the Prince of Wales was brought a good deal into contact with leading medical men may have had something to do with this bent of his mind, but however it arose, questions touching the health of the people seem always to have claimed a large share of his attention.

On the year of his marriage he at once became patron of a considerable number of hospitals, and not in London alone. In 1864 the Prince became a life governor of the London Hospital, giving a hundred guineas to its funds. In 1878 he visited the hospital and gave another hundred guineas. In 1884 he laid the foundation-stone of the new wing, and in 1887 he opened the new buildings, and since then both he and the Princess of Wales have continued to show great interest in this institution, one of the latest instances of which has been the installation by Queen Alexandra of the so-called "light treatment" for cases of lupus, which had been devised by Dr. Finsen, of Copenhagen.

In 1863 the Prince became a patron of the Brompton Hospital, thus, even so early in life, showing an interest in and commencing an association with efforts to control the ravages of consumption, an association and an interest which have of late developed into a serious and widely organised attempt under his presidency to prevent as well as to cure this most fatal malady. In the meantime, however, consumption was with us and had to be treated, and the the old Brompton Hospital proving quite insufficient for the work it had to do, the Prince, in 1879, laid the foundation stone of the new wing by which its accommodation was very largely increased. A few years afterwards he showed his continued interest in the same subject by presiding at a festival dinner in aid of the Royal Hospital for Diseases of the Chest, in the City Road, a festival which handed over nearly £5,000 to the funds, the Prince himself subscribing a hundred guineas. Since that time the problem of consumption has undergone a revolution. New ideas as to its causation have been put forward, and we have begun to understand far more about what is essential for its prevention and in its treatment than entered into the heads of people twenty or thirty years ago. It is then a matter of great interest to observe how quickly the Prince of Wales grasped the altered position of affairs, and at once took a leading part in the crusade against a disease which is the especial curse of the poorer portion of those over whom he now reigns. Indeed, one may say that one of the most striking actions ever taken by the Prince of Wales, and one which shows well how ready he has been to place himself in the van of progress, was his taking up the definite position which he at once assumed in regard to the prevention of tuberculosis. It had long been known that tuberculosis was caused by a microbe—that it was, in truth, an infection. But the universality of the scourge, the long duration of the disease, and the obvious impossibility of shutting up the thousands of people who, although affected with it, were still quite able to do some kind of work, had made men despair of its ever being possible to check the ravages of the malady. A few years ago, however, people began to see that if anything like united action could be taken something might be done, and it is highly characteristic of the Prince of Wales—the author of the celebrated aphorism in regard to typhoid fever, "if preventible why not prevented?"—that as soon as ever it was shown to him that there was hope of doing practical good by making a serious and concerted attack on this terrible disease he gave his name and his influence in furtherance of the great "crusade against consumption" which, during the past two years has done so much to popularise the necessary prophylactic measures. On the formation of a National Association for the Prevention of Consumption and other forms of Tuberculosis, he at once became its president, he has taken an active part in its deliberations, and it is worthy of note that in addition to what he has done in regard to this Association, he is "booked" to preside over a great National Congress on Tuberculosis which will be held in London in April of this year, to which delegates are coming from all parts of the world. It is a striking thing, and shows well how ready the Prince has been to seize the drift of scientific progress, and get at the real heart of things, that at a moment when many, even among the medical profession, were holding back on this question, hardly daring to believe that such a consummation as the prevention of consumption could be possible, the Prince of Wales had the clearsightedness to see that, even if the present movement should not eradicate the disease, it would do an enormous amount of good in many ways, and, therefore, threw himself into the crusade. Thus we may expect to see the great Congress on Tuberculosis presided over by the King of England, not in consequence of this being a duty devolving upon him by virtue of his exalted and responsible position, not as a penalty of office, but as the natural continuation of the good work which he voluntarily took upon himself during the time when, if we may so say, he "was his own master" as the Prince of Wales.

Twice in 1867 the Prince visited St. Bartholomew's Hospital, and on the second of these occasions he became a patron of the institution. On two occasions, again, in 1868 he paid visits to the hospital, and in addition to this he presided at the dinner on May 31st, and was present at the annual inspection in November. In 1879 he opened the new buildings, and on many other occasions, especially in the time of the late Sir James Paget, he paid visits to this great hospital.

At the present day, when so much is done for Londoners by the Metropolitan Asylums Board in the way of providing for those who are laid low with infectious diseases, it is difficult to conceive of the state of affairs when no such institutions were at the disposal of the public. The London Fever Hospital stood almost alone in regard to the treatment of fever cases, and almost all such patients had to be dealt with in the homes of the poor—a disheartening task. In 1863, then the Prince became patron of the London Fever Hospital, and gave a donation to its funds. In 1864 he laid the foundation-stone of the new buildings. In 1877 he gave another donation. In 1878 he visited the hospital. In 1880 he gave a donation. In 1882 he presided at the dinner and gave a hundred guineas. In 1883 he gave another donation of a hundred guineas, and in 1887 he opened some further new buildings which had been erected. But the list of hospitals, general and special, to which the Prince has subscribed, which he has assisted by his presence at festivals, for the buildings of which he has laid the foundation-stones, and which when completed he has opened, is too long to be given here. We must refer our readers to the full list given in a book entitled *Prince, Princess, and People*, by Sir Henry Burdett, K.C.B., published in the year 1890. We would, however, draw attention to the fact that the Prince of Wales, in the interest he took in the hospitals, did not confine his attention to those which were subscription-supported charities. The Prince of Wales has not simply "let himself out," as we have sometimes heard it described, as a mere bait with which to draw subscriptions. He seems to have taken an interest in the sick poor themselves, quite irrespective of this aspect of the question, as was shown on the one hand by his many visits to St. Bartholomew's, an endowed hospital which is independent of the charity of to-day, and on the other by his visits to workhouse infirmaries and his opening of great Poor-law hospitals for the use of those who have come upon the rates.

Now let us turn to later years. On July 12th, 1897, the Prince of Wales, again showing that his interest in hospital affairs extends to hospitals of every kind, opened the Park Hospital, the largest, probably, of the many hospitals erected by the Metropolitan Asylums Board for the treatment of infectious diseases. Replying to an address of welcome, he made a speech on the advantages derived by the community from the possession of isolation hospitals, which showed a complete grasp of the subject, and led the *British Medical Journal* to say, "The Prince's speech is another proof of the interest which he has always displayed in matters relating to the treatment of the sick poor."

On July 19th the Prince of Wales was enrolled an Honorary Fellow of the Royal College of Physicians of London, the ceremony taking place at Marlborough House. "By thus publicly associating himself with one of the great medical corporations the Prince has once more shown the deep personal interest which he takes in matters which so nearly touch the welfare of the people as the maintenance of medical learning and of a high standard of professional zeal and probity." It should be added that the Prince of Wales is the sole Honorary Fellow of the College.

The Prince of Wales has many times visited Guy's Hospital of which he has been president. On May 26th, 1897, he opened the new school buildings, and in so doing made a speech full of common sense on the subject of laboratory work, urging that, although it might be necessary to perform certain experiments with the object of promoting advances in medicine and surgery, the students should nevertheless "cultivate a gentle and humane spirit," which was, he said, the best possession of the medical faculty.

On February 5th the Prince of Wales issued his celebrated letter launching the Prince of Wales's Hospital Fund for London. The occasion was the Diamond Jubilee of Her Majesty the Queen, whose death we all deplore. Many people were wondering in their own minds in what form to commemorate the sixtieth year of her glorious reign, and there was a danger that much money and much loyal goodwill might be frittered away on a multiplicity of objects, some of which might not be such as would be likely to be of permanent utility. The Prince's statement began as follows;—"Having ascertained from the Queen that she has no wish to express a preference for any one of the many proposals loyally suggested for commemorating nationally or locally the sixtieth year of her reign, I feel at liberty to bring to the notice of the inhabitants of the metropolis a project lying very near my heart, its object being to attach the sentiment of gratitude for the blessings which the country has enjoyed during the last sixty years to a scheme of permanent benefaction." "A project lying very near to my heart." That is the way the Prince of Wales spoke of his plan for benefiting the hospitals. And thus was set agoing the great Fund with which, in these latter days, the name of the Prince of Wales has been more especially connected in the public mind, and to the management of which he has devoted a large amount of time and solicitude ever since. The short title is commonly used in speaking of this Fund, but it is worth remembering that the full title given to it by the Prince himself was "The Prince of Wales's Hospital Fund for London to Commemorate the 60th Anniversary of the Queen's Reign." Above all the Prince has set an example of personal service in the cause of humanity. To encourage this he, in 1899, instituted the Order of the League of Mercy. The Order of the League of Mercy, of which Queen Victoria was Patron, is bestowed only upon those who have rendered special personal service in the cause of the hospitals. The League itself was formed to afford opportunities for the collection of small contributions more especially, so that the poorest might join with the richest in rendering help to the hospitals through the Prince of Wales's Hospital Fund.

Thus we see how large a portion of the thought and of the public work of the Prince of Wales has been centred in hospitals and in schemes for lessening the incidence of disease upon the people of this country. And the influence of a prince is not to be measured quite in the same way as that of other people. It is an influence which filters down deep through many unsuspected channels, to break out as springs of charity often many years afterwards; and we cannot but think that much of the care and labour and personal concern in the doings of hospitals, and in questions touching the humane treatment of the sick, whether in hospitals, in workhouses, or in their own homes, which is now given so freely by both high and low on every side and is so striking a characteristic of modern times, to say nothing of the mass of solid cash which has been collected from so many sources during recent years, has had its root in the constant and consistent display of interest in such matters made during so many years by the Prince of Wales. Many a sick sufferer during many years to come will have good cause to rejoice that the King of England is one who, while Prince of Wales, threw in his lot for good and all with the sacred cause of charity.

